# Serum levels of vitamin D, retinol, zinc, and CRP in relation to obesity among children and adolescents

**DOI:** 10.1186/s40001-022-00670-7

**Published:** 2022-04-04

**Authors:** Yan Zou, Ronghua Zhang, Lichun Huang, Dong Zhao, Danting Su, Jia Meng, Yueqiang Fang

**Affiliations:** Zhejiang Provincial Center for Disease Control and Prevention, Hangzhou, China

**Keywords:** Vitamin D, Retinol, C-reactive protein, Obesity

## Abstract

**Background:**

The aim of this study was to examine the possible association between serum micronutrients (vitamin D, retinol, zinc), C-reactive protein (CRP), and obesity among children and adolescents.

**Methods:**

Weight and height were measured and serum 25-hydroxy vitamin D, serum retinol, serum zinc, and CRP were measured in 2818 children and adolescents (6–17 years of age), and the data of 10 investigation sites in Zhejiang Province were used. The difference of micronutrients (vitamin D, retinol, zinc) and CRP among different nutritional status were explored by ANOVA and Chi-square test. The associated factors of micronutrients and CRP of overweight and obesity were explored by multifactor analysis.

**Results:**

There were significant differences between male students and female students on BMI, 25(OH)D3, and CRP, and there were significant differences between children and adolescents and between students living in urban area and rural area on BMI, 25(OH)D3, retinol, and zinc(*t* > 1.96, *p* < 0.05). There were significant differences on 25(OH)D3 and CRP level among children and adolescents with different nutritional conditions (*F* = 2.612, 15.022, *p* < 0.05). In multifactor analysis, we found that female [odds ratio (OR) = 0.68, 95% CI 0.49–0.81], living in rural area (OR = 0.68, 95% CI 0.56–0.82), age (OR = 0.95, 95% CI 0.92–0.98), high CRP concentration (OR = 1.08, 95% CI 1.04–1.12) and appropriate retinol level (OR = 1.32, 95% CI 1.09–1.59) were associated with obesity compared to low/normal BMI.

**Conclusion:**

Gender, living area, age, CRP concentration, and vitamin A status were associated with children and adolescents with overweight and obesity compared to low/normal BMI. More attention in the intervention of overweight and obesity should be paid to boys living in urban areas, and high serum concentration of CRP should also be concerned.

## Introduction

The lancet commented that the prevalence of overweight and obesity in children and adolescents (5–19 years) was increasing [1]. The finding is consistent with other published studies [2]. WHO estimates that worldwide obesity has nearly tripled since 1975 and over 340 million children and adolescents aged 5–19 were overweight or obese in 2016 [3]. In China, the prevalence of obesity also increased rapidly [4, 5].

Obesity can lead to the increase of non-communicable diseases [4–6], especially children who are obese also have more metabolic and cardiovascular risk factors [7]. Children with excess weight are likely to become adults with obesity [8]. Early prevention of overweight and obesity is of great significance to children and their life cycle.

Meanwhile, vitamin A, vitamin D, and zinc deficiencies have been reported among children and adolescents [9, 10]. Because micronutrients play an important role in energy metabolism, it is necessary to study the relationship between micronutrients and obesity. C-reactive protein (CRP), as an indicator of low-grade inflammation, has been considered a possible risk factor for cardiovascular diseases associated with obesity [11], and CRP also plays as a major determinant of central obesity [12]. This study intends to learn the possible association between serum micronutrients, CRP, and obesity among children and adolescents.

## Methods

### Study design

The data of this study are from the China National Nutrition and Health Survey 2016–2017(CHNNS2016–2017). Children and adolescents from 10 investigation sites in Zhejiang Province were selected as participants to form provincial representative sample of Zhejiang Province.

Field investigation, physical examination, and blood specimen collection were conducted between September 2016 and November 2017 in the sampling site. The nutritional status of children and adolescents were evaluated, and their level of micronutrients (vitamin D, Retinol, Zinc), and CRP were measured. The obesity of children and adolescents was the outcomes and the level of micronutrients (vitamin D, Retinol, Zinc) and CRP were the exposures to be explored.

### Sampling method and study population

The data of this study are a part of the China National Nutrition and Health Survey 2016–2017, and that was a cross-sectional survey designed to examine the health and nutritional status of children and adolescents. In the China National Nutrition and Health Survey 2016–2017, stratified multistage probability sampling method was used and there were 10 surveillance sites, including urban and rural area of Zhejiang provincial coverage. According the plan of the survey, at least 280 children and adolescents between 6 and 17 years old in each surveillance site should be interviewed. In this analysis, a total of 2818 participants were included and children and adolescents with concomitant pathological conditions were excluded.

Ethics approval was obtained from the Ethical Committee of Zhejiang Provincial Center for Disease Control and Prevention. All student guardians provided written informed consent after the research protocols were carefully explained to them. Thus, informed consents from the parents/guardians of all participants were received.

### Measurements and data collection

Demographic information was collected by general information questionnaire. Anthropometrical measurements were conducted by well-trained health workers of local community health center who followed a reference protocol recommended by the WHO [13]. Height was measured without shoes to the nearest 0.2 cm using a portable SECA stadiometer, and weight was measured without shoes and in light clothing to the nearest 0.1 kg on a calibrated beam scale. Body mass index (BMI) was calculated as weight (kg)/height (m)^2^. The BMI thresholds for low weight, overweight, and obesity are based on the screening stand since different age and different gender have different thresholds. Overweight and obesity were defined by the BMI cut-off points recommended in “Screening for overweight and obesity among school-age children and adolescents” (WS/T 586-2018) and low weight was defined by the BMI cut-off points recommended in “Screening standard for malnutrition of school-age children and adolescents” (WS/T 456-2014). Blood samples were collected to detect the concentration of retinol, and we judged marginal deficiency and deficiency of vitamin A among children and adolescents by boundary values of serum retinol level in “Method for Vitamin A deficiency screening” (WS/T 553-2017). Blood samples were collected to detect the concentration of 25-hydroxyvitamin D [25(OH)D], and according to standards recommended by the American endocrine association, we judged deficiency and inadequacy of vitamin D among children and adolescents by boundary values of the concentration of 25-hydroxyvitamin D [25(OH)D] [14].

### Statistical analysis

All the data were analyzed with SAS9.4 (SAS Institute Inc., Cary, NC, USA). Continuous variables were present in the form of mean and standard deviation (SD). Category variables were present in the form of number and percentage. The difference of micronutrients (vitamin D, retinol, zinc) and CRP among different nutritional status were explored by ANOVA and Chi-square test. Reported *p* values were not corrected for multiple testing. The associated factors of micronutrients and CRP of overweight and obesity compared to low/normal BMI were explored by multifactor analysis, respectively (Logistic regression, Backward method). A two side *p* < 0.05 was utilized to assess statistical significance.

## Results

### Subject characteristics

Totally, there were 2818 children and adolescents included in this study, and males accounted for 50.2%. The prevalence of low weight, eutrophic, overweight, and obese was 6.7%, 59.2%, 22.9%, and 11.2%, respectively. The serum concentration of 25(OH)D3, retinol, zinc, and CRP were 2.8 ng/mL (SD, 0.7 ng/mL), 2.7 mg/dL (SD, 0.6 mg/dL), 110.9 mg/dL (SD, 42.6 mg/dL), and 1.0 mg/dL (SD, 4.1 mg/dL), respectively.

### Serum levels of 25(OH)D3, retinol, zinc, and CRP characteristics

The BMI, serum concentration of 25(OH)D3, and CRP in males were 18.6 kg/m^2^, 2.9 ng/mL, and 1.2 mg/dL, respectively, and higher than that in females (*t* > 1.96, *p* < 0.001). The serum concentration of 25(OH)D3 and zinc in children was 2.7 ng/mL and 107.0 mg/dL, respectively, higher than that in adolescents(*t* > 1.96, *p* < 0.001). The BMI and serum concentration of retinol in children were 18.7 kg/m^2^ and 2.8 mg/dL respectively, and lower than that in females (*t* > 1.96, *p* < 0.001). The BMI and serum concentration of retinol among children and adolescents living in urban areas were 18.7 kg/m^2^ and 2.8 mg/mL respectively, and higher than those living in rural areas (*t* > 1.96, *p* < 0.001). The serum concentration of 25(OH)D3 and zinc among children and adolescents living in urban areas were 2.7 ng/mL and 107.0 mg/dL, respectively, and lower than those living in rural areas (*t* > 1.96, *p* < 0.001) (Table 1).

**Table 1:** BMI and serum levels of 25(OH)D3, retinol, zinc, and CRP among children and adolescents stratified by gender, age group, and living area

Variables	Total	Gender			Age group			Living area		
		Males	Females	*p*	Children	Adolescents	*p*	Urban	Rural	*p*
BMI (kg/cm^2^)	18.3 (3.8)	18.6 (3.7)	18.1 (3.9)	0.001	16.6 (2.9)	19.5 (3.5)	< 0.001	18.7 (4.1)	18.0 (3.4)	< 0.001
25(OH)D3 (ng/mL)	2.8 (0.7)	2.9 (0.7)	2.7 (0.7)	< 0.001	3.0 (0.7)	2.7 (0.7)	< 0.001	2.7 (0.7)	2.9 (0.7)	< 0.001
Retinol (mg/dL)	2.7 (0.6)	2.6 (0.6)	2.7 (0.5)	0.074	2.5 (0.6)	2.7 (0.5)	< 0.001	2.8 (0.5)	2.6 (0.6)	< 0.001
Zinc (mg/dL)	110.9 (42.6)	112.1 (41.)	109.7 (44.0)	0.148	114.7 (55.5)	108.6 (34.1)	< 0.001	107.0 (28.8)	114.9 (52.8)	0.001
CRP (mg/dL)	1.0 (4.1)	1.2 (5.3)	0.8 (2.3)	0.042	0.9 (2.5)	1.0 (4.4)	0.707	1.1 (4.9)	0.9 (3.0)	0.339

^a^Values in parentheses are SD

There were significant differences on serum concentrations of 25(OH)D3 and CRP among children and adolescents with different nutritional conditions (*F* = 2.612, 15.022, *p* < 0.05). The concentration of 25(OH)D3 and CRP among children and adolescents with obesity were 2.9 ng/mL and 2.4 mg/dL, respectively, higher than those without obesity. (Table 2).

**Table 2:** Serum levels of 25(OH)D3, retinol, zinc, and CRP among children and adolescents with different nutritional status^a^

	Low weight (*N* = 177)	Eutrophic (*N* = 1980)	Overweight (*N* = 304)	Obese (*N* = 297)	*F*^b^	*p*
25(OH)D3 (ng/mL)	2.8 (0.7)	2.8 (0.7)	2.8 (0.7)	2.9 (0.7)	2.612	0.050
Retinol (mg/dL)	2.6 (0.6)	2.7 (0.6)	2.7 (0.6)	2.7 (0.5)	12.309	0.069
Zinc (mg/dL)	108.0 (34.7)	110.9 (46.9)	110.5 (29.8)	113.5 (27.1)	0.671	0.570
CRP (mg/dL)	0.8 (2.7)	0.8 (3.7)	1.1 (2.6)	2.4 (6.9)	15.022	< 0.001

^a^Values in parentheses are SD

^b^*F* is the statistic from ANOVA

Children and adolescents with obesity had higher concentration of 25(OH)D3 and CRP than Children and adolescents with different nutritional conditions had different vitamin A status (*c*^2^ = 12.704, *p* = 0.048), while their vitamin D status was similar (*c*^2^ = 15.443, *p* = 0.079) (Table 3). Trend Chi-square test showed that the higher the vitamin A status, the larger the BMI value (*c*^2^ = 6.886, *p* = 0.009).

**Table 3:** Vitamins D and A status among children and adolescents with different nutritional status

	Vitamin D status^a^				Vitamin A status^b^		
	Deficiency	Inadequacy	Normal	Appropriate	Deficiency	Marginal deficiency	Appropriate
Low weight	3 (1.7%)	61 (34.5%)	83 (46.9%)	30 (16.9%)	11 (6.2%)	49 (27.7%)	117 (66.1%)
Euthophic	37 (2.0%)	656 (35.7%)	850 (46.2%)	297 (16.1%)	78 (4.2%)	478 (26.0%)	1285 (69.8%)
Overweight	7 (2.3%)	110 (36.2%)	138 (45.4%)	49 (16.1%)	14 (4.6%)	65 (21.4%)	225 (74.0%)
Obese	1 (0.3%)	86 (29.0%)	144 (48.5%)	66 (22.2%)	14 (4.7%)	54 (18.2%)	229 (77.1%)

^a^*c*^2^ = 15.443, *p* = 0.079

^b^*c*^2^ = 12.704, *p* = 0.048; trend *c*^2^ = 6.886, *p* = 0.009

### Multifactor analysis for the associated micronutrients and CRP of overweight and obesity

A total of 2818 children and adolescents were included in multifactor analysis, and we found that female [odds ratio (OR) = 0.68, 95% CI 0.49–0.81], living in rural area (OR = 0.68, 95% CI 0.56–0.82), age (OR = 0.95, 95% CI 0.92–0.98), high CRP concentration (OR = 1.08, 95% CI 1.04–1.12), and appropriate retinol level (OR = 1.32, 95% CI 1.09–1.59) were associated with obesity compared to low/normal BMI. We also found that female (OR = 0.58, 95% CI 0.45–0.76), living in rural area (OR = 0.63, 95% CI 0.49–0.82), age (OR = 0.89, 95% CI 0.86–0.94), high CRP concentration (OR = 1.11, 95% CI 1.06–1.16), and appropriate retinol level (OR = 1.55, 95% CI 1.19–2.02) were associated with overweight compared to low/normal BMI (Table 4).

**Table 4:** Multifactor analysis for the associated micronutrients and CRP of overweight and obesity (*N* = 2818)

Factors	*β*	*p*	OR	95% CI for OR	
				Lower	Upper
Multifactor analysis for children and adolescents with overweight					
Gender (female/male)	− 0.538	< 0.001	0.58	0.45	0.76
Living area (rural area/urban area)	− 0.457	0.001	0.63	0.49	0.82
Age	− 0.112	< 0.001	0.89	0.86	0.94
CRP (mg/dL)	0.103	< 0.001	1.11	1.06	1.16
Vitamin A status (appropriate/marginal deficiency and deficiency)	0.439	0.001	1.55	1.19	2.02
Multifactor analysis for children and adolescents with obesity					
Gender (female/male)	− 0.386	< 0.001	0.68	0.57	0.83
Living area (rural area/urban area)	− 0.392	< 0.001	0.68	0.56	0.82
Age	− 0.053	0.001	0.95	0.92	0.98
CRP (mg/dL)	0.078	< 0.001	1.08	1.04	1.12
Vitamin A status (appropriate/marginal deficiency and deficiency)	0.285	0.004	1.32	1.09	1.59

## Discussion

In the present study, gender, age, living area, CRP concentration, and vitamin A status were associated with children and adolescents with overweight and obesity.

In line with our findings, some systematic reviews have reported on growing prevalence of obesity and overweight among children and adolescents. A meta-analysis concluded that the prevalence of obesity in children and adolescents aged 2–19 years in the United States in 2011–2014 was 17.0%, while the prevalence of extreme obesity was 5.8% [15]. A systematic review in Asia reported that the overall prevalence by gender was 7.0% and 4.8% in boys and girls for obesity in children, and 11.7% and 10.9% in boys and girls, respectively, for overweight in children [16]. We found that the prevalence of overweight and obesity was 22.9% and 11.2%, respectively, higher than the previous literature. Similarly, we also found that the prevalence among males were higher than among females. We found higher prevalence of obesity and overweight in urban areas. Conversely, a study in Bangladesh reported that overweight and obesity were associated with rural participants [17]. A study of China base on 1995–2014 revealed that overweight and obesity increased in Chinese children and adolescents, particularly in rural areas [5]. Our study was carried out in 2016–2017, whether there is a long-term trend in the distribution of obesity in urban and rural areas still needs to be further studied.

CRP is produced in the liver, macrophages, and adipose tissues. Elevated serum CRP is associated with obesity among children. A study in Japan reported that CRP increased the risk of obesity in school children [18]. Nappo reported that CRP levels are associated to higher body mass and overweight/obesity risk in a large population of European children. Children with higher baseline levels of CRP were at higher risk of developing overweight/obesity during growth [19]. We confirmed that CRP level was higher in obese or overweight than that in non-obese children, as reported in previous studies. The mechanism lies in that obese children present increased oxidative stress and impaired inflammation and insulin sensitivity, which in turn results in similar impaired endothelial dysfunction and early signs of atherosclerosis [20]. Obesity is a pro-inflammatory state that may predispose patients to acute coronary syndrome characterized by chronic low-grade inflammation resulting in endothelial dysfunction [21]. Thus, CRP plays an important role in the prevention of chronic inflammation associated with obesity in the early stages of life.

Our previous study reported that the prevalence of vitamin A deficiency was 4.5%, and the prevalence of marginal deficiency of vitamin A was 24.7% [22]. At present, the nutritional status of vitamin A is not optimistic. This study found that the retinol level of obese students is higher than that of non-obese children, which may be related to the dietary structure of obese children [23]. Liang reported that vitamin A metabolism may be disordered in obese children, although children with obesity have higher vitamin levels than lean children [24]. Gajewska reported that BMI value may influence the vitamin A status in obese children after therapy [25]. Gajewska suggested there is an occurrence of relationships between vitamin A and oxidized LDL in prepubertal obese children. Vitamin A concentrations is associated with dyslipidemia [26]. The relationship and mechanism between obesity and vitamin A should be further explored in the future study.

Vitamin D can regulate calcium and phosphorus metabolism in the body and promote its absorption, thereby affecting bone calcification. Human vitamin D can be obtained in two ways. One is in the sunlight under UV irradiation, the other is ingested through food. Shaikh reported that children with high BMI showed extremely high prevalence of vitamin D deficiency [27]. Kang reported that during the coronavirus disease-2019 (COVID-19) pandemic, increased childhood obesity and vitamin D deficiencies were observed [28]. Meta-analysis reported that children and adolescents with obesity have higher risk of vitamin D deficiency [29, 30]. Also, a positive association between obesity and lower 25(OH)D serum concentration was found among Chinese adults [31]. Our study found that children and adolescents with obesity had higher concentration of 25(OH)D3, while in the multifactor analysis, vitamin D status was not the influencing factors for children and adolescents with obesity or overweight. The next step of research will further explore the influencing factors of vitamin D status, in order to better explore the relationship between vitamin D and obesity. Further large-scale prospective cohort studies or randomized controlled trials are warranted to explore this association and causality. It is suggested to encourage physical exercise, the reduction of screen time, and healthy eating habits in order to reduce the prevalence of overweight and obesity in children and adolescents and the impact of associated comorbidities, to keep the appropriate micronutrient status [32].

In China, measurement of serum CRP levels is not included in school health checkups, and it is difficult to obtain informed consent for blood sampling from school children and their parents. Thus, few serological surveys have been performed in healthy children. We performed serological tests in the China National Nutrition and Health Survey 2016–2017 and evaluated the relationship between the test results and obesity.

Limitations of the present study should be noted. Since it is a cross-sectional study, causality cannot be established. Obesity and micronutrient status may be related to dietary intake and physical activity, which needs to be further explored in future research. We have measured some parameters related to nutrition in blood in this study and we will do complete blood picture in future studies to facilitate more comprehensive analysis. Limitation also includes generalizability issues and selection bias issues.

## Conclusions

In summary, the present study documented gender, living area, age group, CRP concentration, and vitamin A status were associated with children and adolescents with overweight and obesity. More attention in the intervention of overweight and obesity should be paid to boys living in urban areas, and high serum CRP level should also be concerned.

## Data Availability

The supporting data can be acquired via correspondence author.
